# Royal Westminster Ophthalmic Hospital

**Published:** 1830-04

**Authors:** 


### HOSPITAL REPORTS.

### ROYAL WESTMINSTER OPHTHALMIC HOSPITAL.

*Report of some of the Cases treated at this Hospital.*

Surgeon, G. J. Guthrie, F.R.S.

### *Rheumatic Inflammation of the Sclerotic, with Iritis. Efficacy of Turpentine.*

December 24th, 1829.—Mary Mallison, æt. thirty-five, a single person, of rather plethoric habit, living as cook in a family, was attacked with rheumatism of the knees and shoulders three months since, but, by the aid of medicine, perfectly recovered. She was enjoying perfect health, when, twelve days since, she was exposed to the night air and thick fog, and the same evening complained of pain and dimness of the left eye, which in the course of a few hours became red, and discharged tears. The right eye was slightly affected; and they both continued bad for seven days, the left being the worse. No application but warm water. On the fourth morning the left was better, and the right became the more affected one, and has continued worse than the left since. She applied three leeches to the right eye, and one to the left, and remained rather better till the ninth evening, when the leeches were reapplied without much benefit. During her illness she has suffered from violent pain in her head, generally worse towards evening; and, for the last few mornings, she has complained of "dreadfully shooting pains through the ball of the right eye, lasting sometimes for two hours."

She is able to bear the light for a short time; does not suffer from much lachrymation, and has no secretion in a morning. The right eye is considerably inflamed; a zone of pink vessels running in straight lines around the cornea, leaving a white line between them and the latter tunic. There are some large tortuous vessels of the conjunctiva, but the inflammation is chiefly of the sclerotic. Cornea clear; aqueous humor turbid; iris oval, of a brownish colour; sight excessively dim; pains in the forehead and eyeball violent towards the evening and early in the morning; furred tongue; confined bowels; pulse 110, strong.—℞. Antimon. Tart: gr. iv.; Aq. puræ ℥iss. fiat haustus st. sumendus.—℞. Sp. Tereb. ʒi. in formâ misturæ post horas quatuor, et rep. quartis horis.—℞. Pulv. Ipecac. co. gr. x. horâ somni sumend.

Dec. 25th.—The draught speedily produced vomiting, which lasted for some time. Bowels most fully acted on; and the surface of the body in a state of profuse perspiration. Took the turpentine at five o'clock, and the powder at bedtime. Passed a good night, and has been free from pain in the head; but has suffered from "throbbing, or rather a burning, in the ball of the eye." Still a good deal of cloudiness; vessels less pink; sight the same; pulse reduced. Has suffered no inconvenience from the turpentine.—Pers. in usu medicament.; i. e. Sp. Tereb. et P. Ipec. co.

27th.—Suffered from severe darting pains through the head and eye, during the night. This morning is relieved, and finds her sight stronger. Pupil more regular; less injection of the sclerotic. Complains of pain and heat in the parts when the urine is voided. Tongue cleaner; bowels open. Forgot to take her powder.—Rep. pulv. et mistura.

28th.—Suffered for three hours this morning with violent pain. Iris more irregular; vessels of the conjunctiva more vascular.—Empl. Belladon. tempori. Rep. med.

30th.—Went into the country yesterday, finding herself much better. Experienced great relief from the belladonna. Has had no return of pain in the head. Has slept well without the powder. Bowels open; still suffers pain when making water. Pupil dilated and more regular. Still has dimness, and a congested state of the conjunctiva.

January 1st.—Has omitted her medicine these two days. Passed a most restless night. Pain in the head returned with violence; conjunctiva more inflamed, constant lachrymation; some intolerance of light; pupil irregular. Fancies she has caught cold.—Rep. mist., pulv., et empl. Belladon.

3d.—Has taken her turpentine every four hours. Bowels gently acted on. No strangury. Has taken her powder at bedtime. No return of pain. Says she is now greatly better than she ever has been since admission; can see considerably better; no lachrymation or intolerance; pupil more regular, conjunctiva less injected. —Repet. medic.

6th.—Had no medicine yesterday. Good night, free from pain. Can see equally well with both eyes; vessels resumed their natural appearance. Considers herself perfectly well. Passes her water without difficulty.—Desired to omit her medicine, and repeat it in two days' time.

9th.—No return of pain, and follows her business as usual.

Called at the hospital a week after, to inform us she was perfectly well.

Although this case was decidedly benefited by the turpentine, and indeed ultimately cured by it, yet it is most probable that, in the first instance, its effects were very much increased by the powerful dose of antimony. The turpentine affected the urethra on the second day, when the eye also was much benefited. She neglected its use, was worse, and repeated her medicine with greater relief than before, without its having any effect on the urinary organs; but, as she was suffering from the catamenia at this period, the turpentine continued to keep up a discharge from the uterus for some days. The bowels were very little acted on by the medicine. The quantity of urine secreted as usual, but depositing considerable sediment.

As an addition to the list of medicinal agents in the cure of several affections of the eye, (more particularly syphilitic iritis,) turpentine is likely to prove a very useful and valuable one; but in the present case it is probable that equally good effects might have been accomplished by a less severe remedy, for the patient appeared much reduced by the effects of the medicine.

### *Syphilitic Iritis.*

Thomas Harper, æt. nineteen; admitted February 9th, 1830. About twelve months since, caught the venereal disease for the first time; had a chancre; applied the black wash, and took pills, which he says did not make his mouth sore. An eruption appeared on his body about two months after the chancre healed: for this he took some medicine which made his mouth sore, and which he thought cured him, as the disease in great measure disappeared. Five months since had gonorrhœa, but was soon cured, and continued well till six weeks since, when his right eye became rather dim and very painful. This continued for a few days, when the pain disappeared; but it returned about two days since, as violent as before, with increased dimness, amounting almost to total blindness. In this state he was admitted this morning: iris of the right eye irregular, contracted, and almost stationary, with discoloured surface.—Gutt. Belladon. ℞. Pulv. Jalap. co. ℈i. cras mane. ℞. Sp. Terebinth. ℈i. ter die.

11th.—Pupil more regular; colour of the iris improved, as also vision.—Rep. med.

13th.—Going on well.

17th.—The eye has continued gradually to improve; irregularity of iris nearly gone. Has passed blood from the urethra.—Ordered to discontinue the medicine. ℞. Pil. Hydrarg. gr. v. h. s. Sulph. Magnes. ℥i. mane.

19th.—Convalescent. Makes water well.—Ordered to return to his medicine.

23d.—The lad is quite well. The eruption has disappeared; vision perfect; eye resumed its natural appearance.

